# COMBATdb: a database for the COVID-19 Multi-Omics Blood ATlas

**DOI:** 10.1093/nar/gkac1019

**Published:** 2022-11-10

**Authors:** Dapeng Wang, Vinod Kumar, Katie L Burnham, Alexander J Mentzer, Brian D Marsden, Julian C Knight

**Affiliations:** Wellcome Centre for Human Genetics, University of Oxford, Oxford OX3 7BN, UK; Kennedy Institute for Rheumatology, University of Oxford, UK; Wellcome Sanger Institute, Cambridge, UK; Wellcome Centre for Human Genetics, University of Oxford, Oxford OX3 7BN, UK; Kennedy Institute for Rheumatology, University of Oxford, UK; Centre for Medicines Discovery, NDM, University of Oxford, Oxford, OX3 7BN, UK; Wellcome Centre for Human Genetics, University of Oxford, Oxford OX3 7BN, UK; Chinese Academy of Medical Science Oxford Institute, University of Oxford, UK; NIHR Oxford Biomedical Research Centre, Oxford, UK

## Abstract

Advances in our understanding of the nature of the immune response to SARS-CoV-2 infection, and how this varies within and between individuals, is important in efforts to develop targeted therapies and precision medicine approaches. Here we present a database for the COvid-19 Multi-omics Blood ATlas (COMBAT) project, COMBATdb (https://db.combat.ox.ac.uk). This enables exploration of multi-modal datasets arising from profiling of patients with different severities of illness admitted to hospital in the first phase of the pandemic in the UK prior to vaccination, compared with community cases, healthy controls, and patients with all-cause sepsis and influenza. These data include whole blood transcriptomics, plasma proteomics, epigenomics, single-cell multi-omics, immune repertoire sequencing, flow and mass cytometry, and cohort metadata. COMBATdb provides access to the processed data in a well-defined framework of samples, cell types and genes/proteins that allows exploration across the assayed modalities, with functionality including browse, search, download, calculation and visualisation via shiny apps. This advances the ability of users to leverage COMBAT datasets to understand the pathogenesis of COVID-19, and the nature of specific and shared features with other infectious diseases.

## INTRODUCTION

Deep immune phenotyping including multi-omic profiling at single cell resolution advances our ability to understand the host response to infection, and how this may both enable recovery and where dysregulated, contribute directly to pathogenesis (1–5). Such profiling has been applied at unprecedented scale and depth during the COVID-19 pandemic. Technologies employed have included high resolution flow and mass cytometry, single cell transcriptomics, proteomics and epigenomics, proteomics, and metabolomics, with application primarily to blood (6–20) but also autopsy and lung samples (21,22), bronchoalveolar lavage (23,24), nasopharyngeal and bronchial sampling (25). Such data can provide insights into mediators and processes driving disease, identify potential therapeutic targets and advance understanding of why some people become severely ill, highlighting opportunities for precision medicine (4,26). Together with clinical and experimental approaches, this has advanced our understanding of disease pathogenesis in COVID-19 and in particular the nature of local and systemic immunopathology and dysregulation driving severe disease (27,28). Here, we present COMBATdb (https://db.combat.ox.ac.uk), a database to explore datasets arising from one such programme of work, the COvid-19 Multi-omics Blood ATlas (COMBAT) Consortium (8).

COMBAT recruited participants without prior vaccination or exposure history at the start of the pandemic in the UK, before widespread use of dexamethasone for severe disease, providing a unique viewpoint on the host response state. Individuals with COVID-19 were compared between those in the community and hospitalised cases with mild, severe and critical illness. It remains unclear the extent to which mechanisms are shared between patients with COVID-19 who develop life threatening organ dysfunction and those with all-cause sepsis and with critical illness arising from viral infections, notably influenza (29,30). COMBAT provides datasets allowing comparison with these groups, and with healthy controls. The COMBAT datasets span a breadth of modalities linked to a given blood draw (sample), including whole blood mass cytometry and transcriptomics (total RNA-Seq); B and T cell repertoire sequencing; peripheral blood mononuclear cells (PBMC) assayed using single-cell RNA and repertoire sequencing with epitope measurements (Cellular Indexing of Transcriptomes and Epitopes by Sequencing, CITE-Seq), chromatin accessibility (scATAC-Seq) and flow cytometry; and plasma proteomics (timsTOF mass spectrometry and Luminex immunoassays). COMBATdb provides a web application upon a relational database to explore and access these datasets, specifically processed data and associated metadata, with tools to browse, calculate, and download results, together with visualisation via shiny apps.

## DATABASE CONTENTS

**Figure 1.**
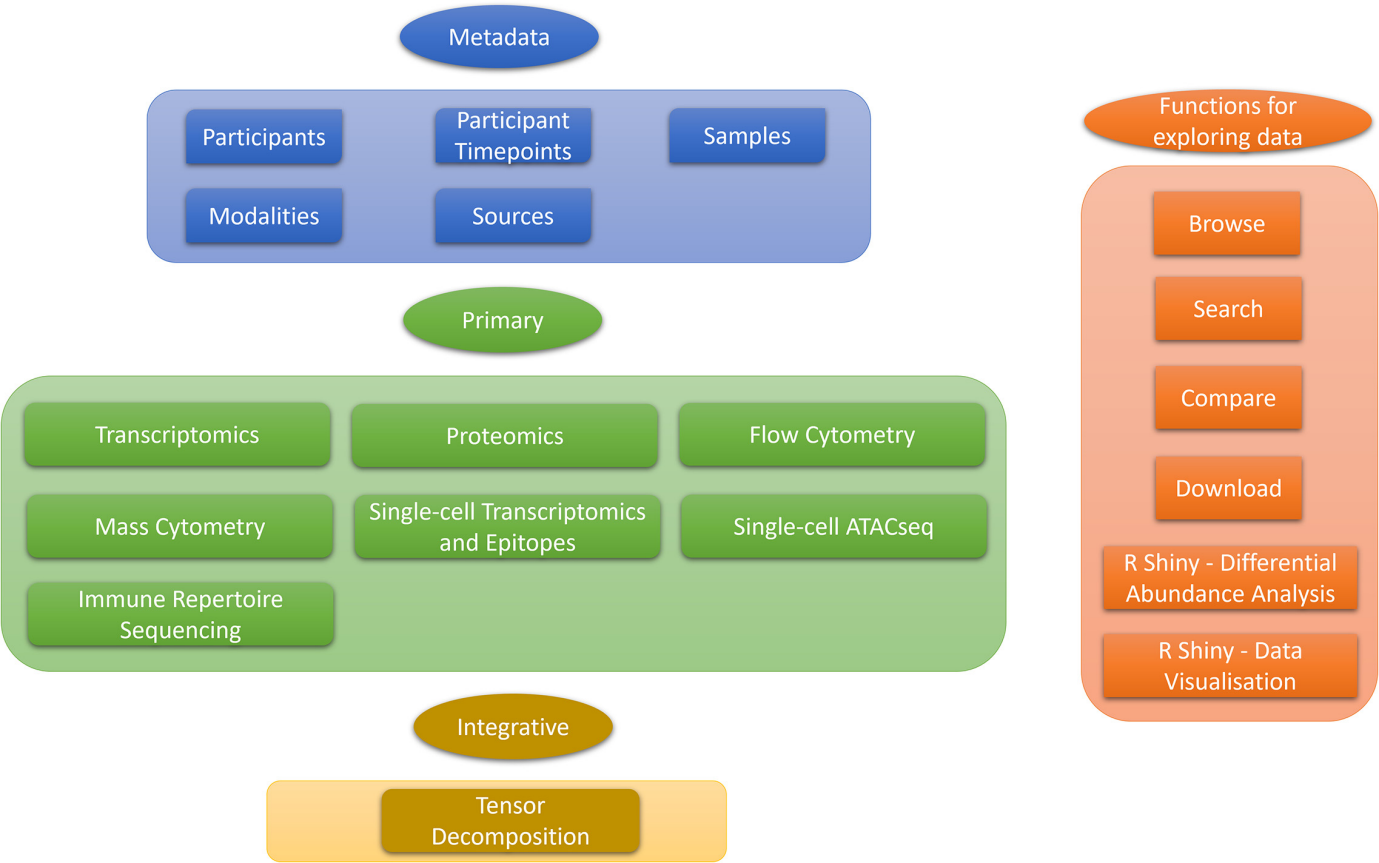
Schematic summarising the main modules of COMBATdb.

COMBATdb contains the metadata, immune phenotyping and omics data reported by the COMBAT Consortium with a user-friendly interface to query, investigate and visualise the data (Figure 1). All data arising from any assay modality are organised through the unified COMBAT sample identifier system and can be linked via the sample IDs. Metadata for assay modalities, participants, samples, sampling timepoints, and processing are provided together with their connectivity. The database allows query entry points through specific cell types, genes/proteins and samples or through each assay modality. COMBATdb provides pre-calculated differential abundance analysis results based on optimised parameters, and supports real-time calculation for comparison between groups of samples based on the user-inputted options.

## METADATA: PARTICIPANTS, TIMEPOINTS, SAMPLES, MODALITIES AND SOURCES

**Figure 2.**
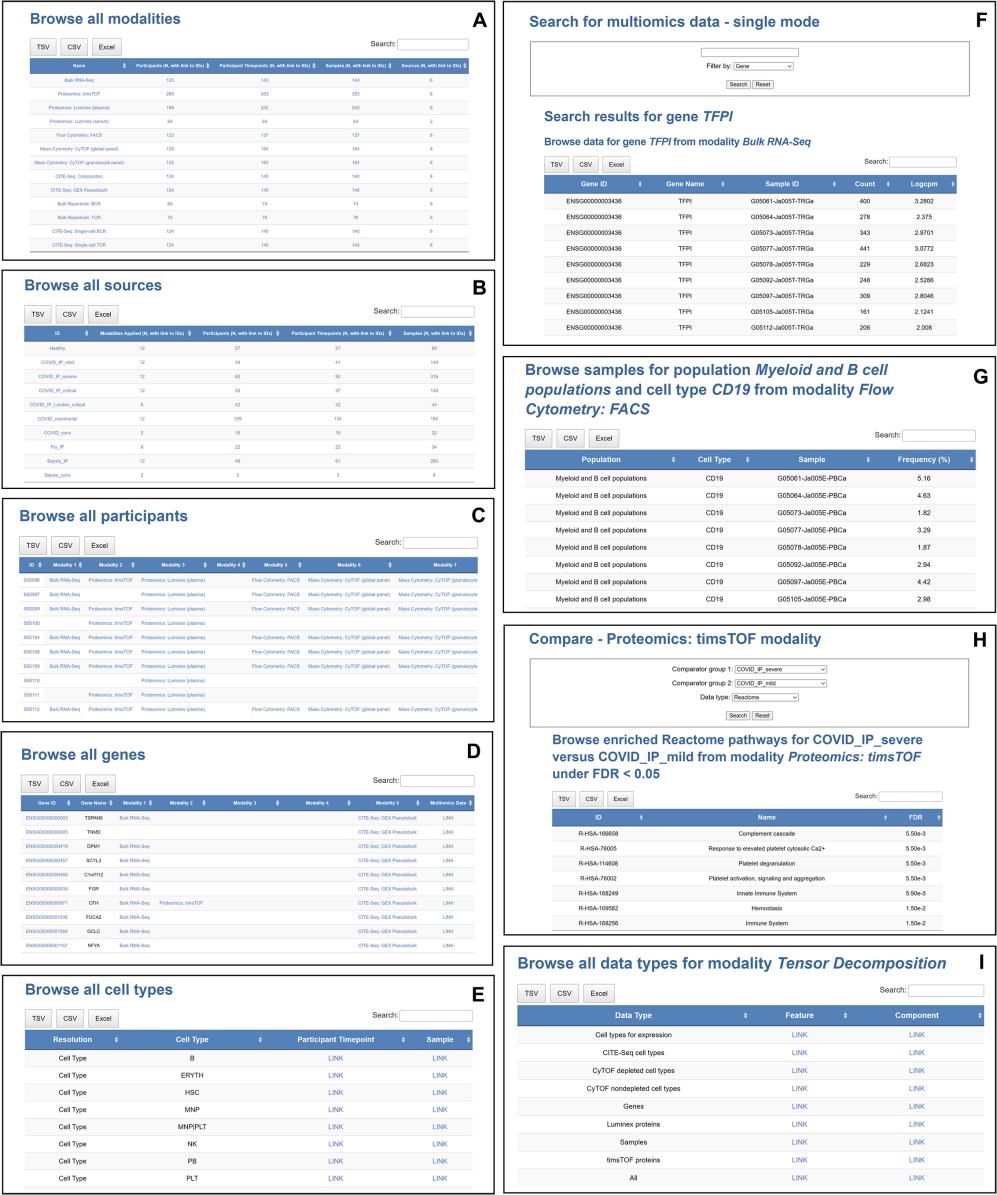
Examples of COMBATdb pages. (A–E) Lists of browsable (**A**) assay modalities (**B**) sources (comparator groups) (**C**) participants (**D**) genes (**E**) cell types. (**F**) Landing page for multiomics data single search mode and the results. (**G**) Example of results for samples from Flow Cytometry: FACS modality. (**H**) Landing page for compare functionality and the resulting enriched Reactome pathways for Proteomics: timsTOF modality. (**I**) List of browsable features and components for various data types on the landing page of tensor decomposition modality.

The five key terms relating to the metadata in COMBATdb are ‘participants’, ‘participant timepoints’, ‘samples’, ‘modalities’, and ‘sources’. We established the COMBAT patient and sample identifier system to ensure that all essential information was contained in the sample ID itself, including cohort, participant ID, recruitment site, sub-sample ID, sample type, derived sample type, processing site and batch (8). Based on this, the participant timepoint IDs and participant IDs can be deduced from the sample IDs, and all are robustly linked. ‘Modalities’ refer to datasets generated from the same type of samples (e.g. plasma), produced through the same assay platform (e.g. Luminex immunoassay) and analysed in the same data analysis workflow—in this example, giving rise to the ‘Proteomics: Luminex (plasma)’ modality (Figure 2A). For some platforms, such as single cell transcriptomics/proteomics assayed using CITE-Seq, there are multiple modalities arising following application to PBMCs: gene expression counts combined at minor subset, major subset and cell type level (‘CITE-Seq: GEX Pseudobulk’ modality); cell counts at minor subset, major subset and cell type level (‘CITE-Seq: Composition’ modality); B cell repertoire (‘CITE-Seq: Single-cell BCR’ modality) and T cell repertoire (‘CITE-Seq: Single-cell TCR’ modality). We have currently included one integrated modality (tensor decomposition) that was derived from cellular composition, gene expression, and plasma proteomics (8) using a sparse decomposition of arrays (SDA) algorithm (31) with loading scores and/or posterior inclusion probabilities available. The data from each of the 13 modalities has been processed through the specific pipelines of the primary analysis as described in the STAR Methods section of the published COMBAT paper (8) following best practice with optimal parameters, state-of-the-art tools and consensus annotations. In order to meet the broad range of requirements of using the data, different types of normalised data have been stored in the database for the same modality or different modalities (Table 1). ‘Sources’ refers to the comparator group to which that sample belongs, including healthy volunteers, community COVID-19 cases, COVID-19 hospitalised mild, severe or critical, convalescent sampling from hospitalised cases, critical cases with influenza and all cause sepsis (hospitalised and convalescent) (Figure 2B).

**Table 1.** The modalities and their associated data types

Modality	Data type
Bulk RNA-Seq	count, logcpm
Proteomics: timsTOF	intensity
Proteomics: Luminex (plasma)	fluorescence intensity, concentration
Proteomics: Luminex (serum)	fluorescence intensity, concentration
Flow Cytometry: FACS	cell frequency
Mass Cytometry: CyTOF (global panel)	cell frequency
Mass Cytometry: CyTOF (granulocyte panel)	cell marker expression
CITE-Seq: GEX Pseudobulk	count, residual, RPM
CITE-Seq: Composition	cell count, cell frequency
Bulk Repertoire: BCR	BCR metrics
Bulk Repertoire: TCR	TCR metrics
CITE-Seq: Single-cell BCR	chain locus, contig QC
CITE-Seq: Single-cell TCR	chain composition

## FUNCTIONS FOR EXPLORING DATA

The ‘Browse’ function enables the user to list all the entries of each metadata term (modalities, participants, participant timepoints, samples and sources) and explore linked information about other metadata terms related to the selected entry. We provide two advanced ways of exploring the samples on the sample details page. The first shows the breakdown of sample attributes (for example cohort) that have been stored in the database. The second displays all samples that possess the specific attribute. COMBATdb supports the browsing of key processed data from the major modalities for participants, participant timepoints and samples, allowing the users to interrogate the various types of data from the same sample, participant timepoint or participant. For example, for each participant, participant time point and sample users can rapidly have an overview of which of the 13 individual modalities are available and browse them (Figure 2C). In addition, we also provide ‘genes’ and ‘cell types’ as browsable terms (Figure 2D, E). Specifically, the ‘genes’ function can link five different modalities where gene or protein expression can be measured and calculated, such as ‘Bulk RNA-Seq’, ‘Proteomics: timsTOF’, ‘Proteomics: Luminex (plasma)’, ‘Proteomics: Luminex (serum)’ and ‘CITE-Seq: GEX Pseudobulk’. The cross-referencing between transcriptomics and proteomics data was performed based on shared gene names. The three levels of resolution of cell types (‘Cell type’, ‘Major cell subset’ and ‘Minor cell subset’) include data from ‘CITE-Seq: GEX Pseudobulk’ and ‘CITE-Seq: Composition’.

The ‘Search’ function supports search by metadata in three modes: single, multiple and customisable. For single and multiple mode, the search results will return detailed information about single or multiple terms from modalities, participants, participant timepoints, samples, sources, genes and cell types. Using the customisable mode generates a list of samples, participant timepoints or participants that are defined by a given modality and a given source. For multiomics data, ‘single mode - multiomics data’ searches for a single participant, participant timepoint, sample, gene or cell type and returns the results of multiomics data on the same page, which enhances the comparison capability of cross modality data (Figure 2F). The ‘cell type’ page will display the cell types that are associated with the five modalities involving specific cell types arising from flow cytometry, mass cytometry and CITE-Seq (‘Flow Cytometry: FACS’, ‘Mass Cytometry: CyTOF (global panel)’, ‘Mass Cytometry: CyTOF (granulocyte panel)’, ‘CITE-Seq: GEX Pseudobulk’ and ‘CITE-Seq: Composition’).

The ‘Primary’ function offers a way to access the data from each modality with different layouts for different modalities of data (Figure 2G). For gene/protein-oriented modalities, the landing page will be a selection of genes/proteins, and the user can further navigate to see the expression values for all samples for the gene/protein of interest. For the modalities involved with cell types, the user can choose the cell type first and then explore the data for all samples from a specific cell type. ‘CITE-Seq: GEX Pseudobulk’ is an exceptional case where both genes and cell types are present, and the data are organised in the order genes to cell types to samples.

The ‘Compare’ function provides pre-calculated differential abundance analysis tables across two comparator groups of samples under the cut-offs of FDR < 0.05 and fold change > 1.5 for 7 out of 8 modalities. More stringent cut-offs (FDR < 0.01 and fold change > 2) have been applied for the ‘CITE-Seq: GEX Pseudobulk’ modality. For some of the modalities, this function offers functional analysis using Gene Ontology (32,33) terms and Reactome pathways (34) under FDR < 0.05 (Figure 2H). In particular cases, two source groups can be further combined to form a new source group such as ‘COVID_IP_severe_and_COVID_IP_critical’ that represents the merged group of ‘COVID_IP_severe’ and ‘COVID_IP_critical’.

The ‘Integrative’ function provides a high-level picture of multimodal data analysis integration. The ‘Tensor decomposition’ module offers two ways of starting to query the data, through either feature or SDA component. Each component comprises vectors of scores (loadings) indicating the contribution of individual features (cell types, genes, or proteins) linked by that component (Figure 2I). This design allows the user to compare loading scores and/or posterior inclusion probabilities for different features for the same component and data type, or for different components for the same feature and data type.

The ‘Download’ function provides two tables that contain information such as dataset ID, description and accession number as well as links to download the key processed datasets for this database and the COMBAT project more broadly, and how to access raw datasets.

## SHINY APPS

We have prepared shiny apps (using the R shiny package) for a selection of modalities to allow differential abundance analysis and data visualisation.

The differential abundance analysis module accepts the user-provided options of two comparator groups (case versus control), sample inclusion strategy, covariates, FDR threshold and fold change threshold. The results are in the form of tables, volcano plots and boxplots. We provide three different strategies for inclusion, namely ‘all samples’, ‘priority samples’ (the first sample available at the maximum sampled severity for each patient) and ‘priority samples at maximum severity’ (first sample taken at the maximum clinical severity for that patient) to give user flexibility in the analysis. Covariates such as ‘age and sex’, ‘age’ and ‘none’ are adjusted for in the differential abundance analysis by defining the appropriate design matrix to fit the linear model in edgeR package (35) or limma package (36) except for ‘Proteomics: Luminex (plasma)’ and ‘Proteomics: Luminex (serum)’ where we use t-test, and for ‘Bulk Repertoire: BCR’, ‘Bulk Repertoire: TCR’ and ‘CITE-Seq: Single-cell TCR’ the Wilcoxon test. Although the data in the differential abundance table are calculated from the selected two groups of samples, the boxplots can be used to visually assess the abundance pattern across all source groups and direction of abundance changes for a specific gene, protein or cell cluster. For the cell type relevant modalities, we also provide the options of cell population or cell group resolution to add more levels to explore the data. To increase the readability of the name labels, we only show the top 20 most significant genes, proteins or cell clusters in each volcano plot.

For the data visualisation module, we adopted high dimensional reduction techniques to generate principal component analysis (PCA) plots, correlation plots, loadings plots and heatmaps based on the user defined dimension for X axis, dimension for Y axis, sample inclusion strategy and covariates. Only source groups that contain at least 3 samples have been retained for further analysis and are displayed in the plots. For ‘Flow Cytometry: FACS’ and ‘Mass Cytometry: CyTOF (global panel)’, we use the CATALYST package (37) to prepare the data that are used to produce the PCA plots and loadings plots. For other modalities, we exploit the prcomp function and ggplot2 package to compute the PCA and make the PCA plots. To improve the visibility of labels on loadings plots, we only display the gene names for the points with larger loading scores in at least one of the two axes. For the tensor decomposition analysis, users can visualise the data by selecting the component of interest and choosing the posterior inclusion probability and two types of charts (boxplots and barplots) will be generated accordingly. The boxplot shows the general trends of each patient source group in terms of loading scores while the barplot for cell types for expression indicates the loading scores for whole blood and eight cell types (haematopoietic stem (and progenitor) cells, plasmablasts, natural killer cells, platelets platelet/CD34- megakaryocyte progenitor cells, B cells, T cells, mononuclear phagocytes, erythrocytes). The remaining barplots present the loading scores of cell types, proteins and genes whose corresponding posterior inclusion probability is no less than the given cut-off in an ascending order. In particular, only the bottom 50 genes and top 50 genes according to their loading score rank are shown for the gene barplot to ensure the visibility of gene names. Collectively, this module will support observation of the patterns of each source comparator group of samples and identification of the extent of contribution of each feature to each component.

## IMPLEMENTATION

In anticipation of further data and modalities becoming available as part of COMBAT using existing or new samples, we adopted a modular and scalable philosophy in designing and developing COMBATdb. For the database schema, we created a number of MySQL tables to contain the metadata and modality data in a highly efficient manner. In brief, we have separate tables for modality, participant, participant timepoint, sample, source, gene and cell type. The key linker table is the many-to-many ‘modality2sample’ table. Each modality has an independent table for ‘primary’ and ‘compare’ function. To expedite data queries, indices have been created on certain columns of the data table. To mitigate against SQL injection security risks, we employed prepared statements throughout the platform. All the tables in the interface are searchable, sortable, and downloadable. To improve the performance of R shiny apps, we stored the input data in R object files that have been sufficiently processed and, in this way, the response time of running shiny apps is significantly reduced to an acceptable time period.

### Use case— exploring datasets to identify novel biomarkers

**Figure 3.**
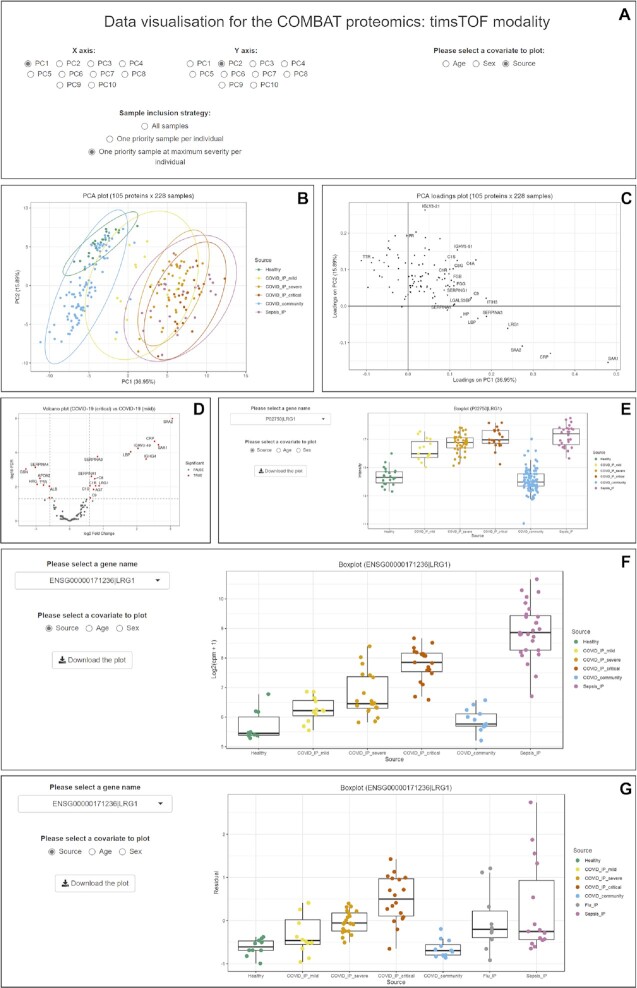
Examples of analysis modules in shiny apps. (A–E) Plasma proteomics: timsTOF modality (**A**) options on the visualisation module (B, C) analysis generated from ‘One priority sample at maximum severity per individual’ (**B**) principal component analysis (PCA) plot coloured by source comparator groups (**C**) PCA loadings plot labelled by protein names. (**D**) Volcano plot based on the results from differential abundance analysis between COVID-19 (critical) and COVID-19 (mild). (**E**) Boxplot for the protein expression levels of a given protein (LRG1) grouped by source comparator groups. (**F**) Boxplot of LRG1 from ‘Bulk RNA-Seq modality’. (**G**) Boxplot of LRG1 from ‘CITE-Seq: GEX pseudobulk modality’ with cell group resolution ‘cell type’ and cell cluster name ‘MNP’ (mononuclear phagocytes).

Here we briefly illustrate an example of how the data can be explored within and across the multi-omic datasets. An important research question is the identification of potential biomarkers in COVID-19 for more severe disease amongst hospitalised patients. Plasma proteins are often used for point of care testing in other disease settings. A global overview of the major components of variance in all assayed plasma proteins comparing all participants is possible by principal components analysis using the ‘Visualisation’ function for timsTOF (Figure 3A). This shows separation along the largest component of variance (PC1) by comparator group (broadly a gradient between healthy, mild and most severely ill patients) with a number of proteins of potential interest contributing to PC1 on the PCA loadings plot. For example the adipokine leucine rich alpha-2 glycoprotein (LRG1) has the fourth highest loading score after serum amyloid proteins and C-reactive protein (CRP) (Figure 3B, C). This can also be explored directly within the timsTOF modality using the shiny app, generating a volcano plot for all assayed plasma proteins comparing hospitalised COVID-19 patients with critical vs mild disease (Figure 3D). This shows that LRG1 is more abundant in critical COVID-19 patients (FDR = 0.009 and log_2_FC = 0.88), consistent with its role in innate immunity including as an inducible upstream modulator of TGFβ signalling (38).

**Figure 4.**
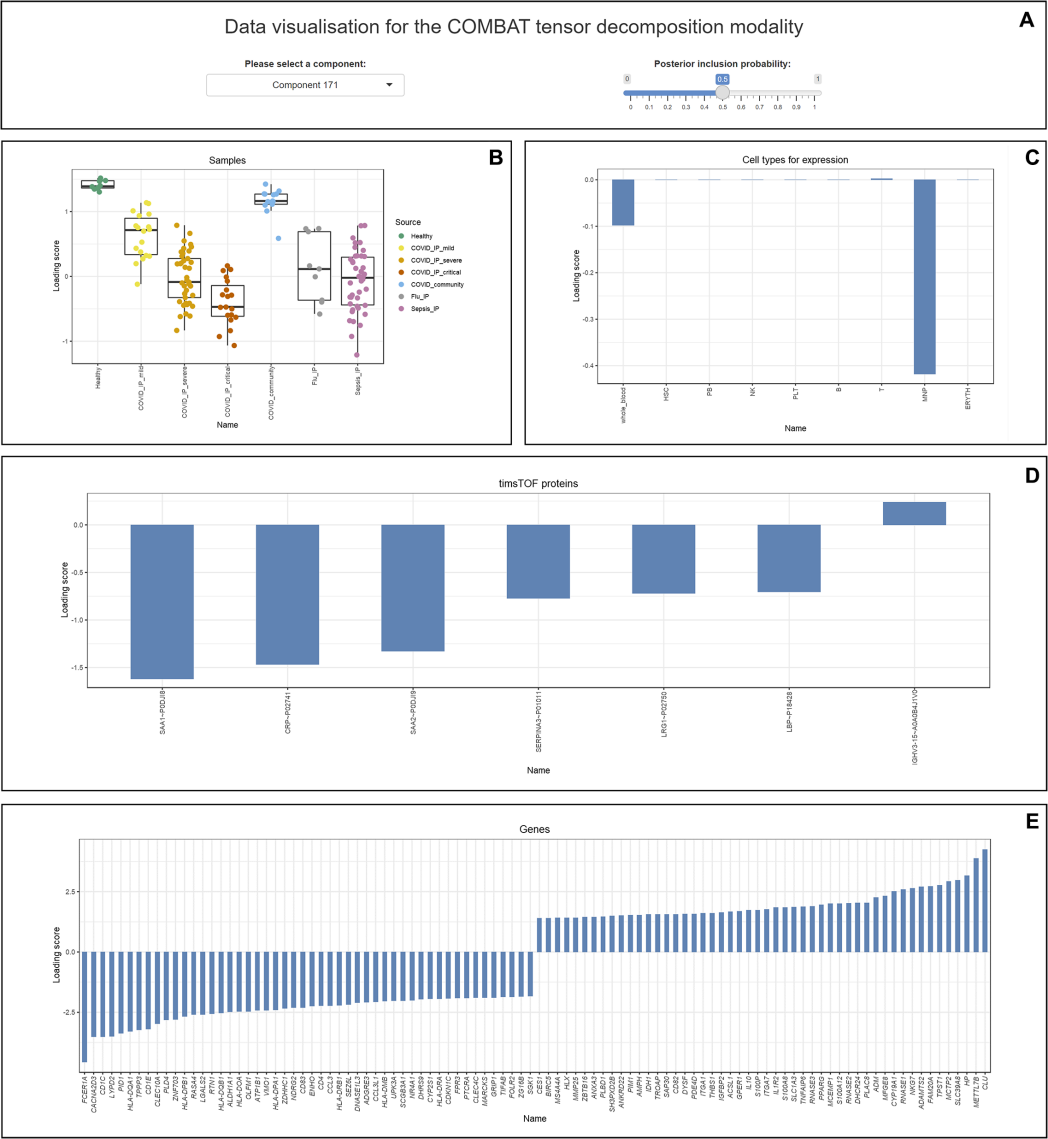
Integrative analysis using tensor decomposition. (**A**) Options within the visualisation module of the tensor decomposition modality. (B–E) Tensor decomposition shiny app showing (**B**) boxplot of loading scores of samples grouped by comparator group sources (**C**) barplot of loading scores for nine cell types for expression (**D**) barplot of loading scores for timsTOF proteins (**E**) barplot of loading scores of genes.

LRG1 protein abundance can also be visualised using box plots across COVID-19 critical and mild patients, and other comparator groups (Figure 3E). To explore this protein further at a multi-omic level, the total RNA-Seq of whole blood dataset can be interrogated by the shiny app. This shows differential expression of *LRG1* between COVID-19 critical and mild cases (FDR = 0.00003 and log_2_FC = 1.51) (Figure 3F) which can be resolved to specific cell types form the single cell CITE-Seq data, for example mononuclear phagocytes (FDR = 0.008 and log_2_FC = 1.38) (Figure 3G). The different omic datasets can also be combined and interrogated using tensor decomposition. This shows that component 171 is most strongly associated with COVID-19 severity (8) and further highlights LRG1. The component can be visualised using the shiny app (Figure 4A) including box plots of loading scores by source comparator group (Figure 4B), with contributing cell types to the tensor component in terms of gene expression involving mononuclear phagocytes and to a lesser extent whole blood (Figure 4C), plasma proteins including SAA1/2, CRP, SERPINA3 and LRG1 (Figure 4D) and differential gene expression (Figure 4E).

This example highlights the way the COMBAT datasets can be explored within COMBATdb, and a particular example involving LRG1 which adds to evidence for its potential utility as a biomarker and therapeutic target in a variety of diseases (38).

## CONCLUSIONS

Multi-omics and associated deep immunological profiling generate substantive high dimensional datasets, requiring careful attention to data management and informatics to allow effective databasing and maximise downstream utility of datasets. On behalf of the COMBAT Consortium, we have presented COMBATdb, a relational database that includes data across modalities generated from a unified set of samples. COMBATdb utilises key terms such as samples, genes/proteins and cell types to organise the various types of data in a user-friendly manner. It utilises cutting-edge bioinformatics tools such as R shiny apps together with other mainstream R packages to establish a flexible, intuitive and interactive visualisation platform that includes a set of user-provided options compatible with the experimental design and intrinsic data structure. The datasets in COMBATdb have been processed and standardised by following the best practice in each omics domain, the organisation of datasets in each modality supporting reanalysis and exploration of specific scientific questions. COMBATdb provides different approaches to explore the datasets from different angles to validate or complement the results of single analysis, including the integrative analysis of multiple omics datasets. Further interactive data visualisation is available at https://mlv.combat.ox.ac.uk/ (8). Future work will incorporate new assays on existing and new samples, and look to support meta-analysis involving comparable studies. A variety of databases and webservers leverage multi-omics data in diverse areas of disease and biology including aging processes (39), cancer (40,41), biological networks (42), human microbiome (43), disease associations (44), host responses to viral infection (45) and specifically COVID-19 (46,47). We envisage that COMBATdb, as part of the COMBAT project (8), will support investigation of COVID-19 and other infectious diseases, and hope that the framework built here will facilitate the development of other multi-omics databases.

## DATA AVAILABILITY

COMBATdb is hosted at https://db.combat.ox.ac.uk.

Details of accession numbers for deposited data at Zenodo (https://doi.org/10.5281/zenodo.6120249) and European Genome Phenome Archive (EGAS00001005493) and ProteomeXchange Consortium via the PRIDE partner repository (PXD023175) are provided within the source publication (8) and COMBATdb.

## Supplementary Material

gkac1019_Supplemental_File

## Notes

Present address: Dapeng Wang, National Heart and Lung Institute, Imperial College London, London, SW3 6LY, UK.
