# Supplementary material for: COMBATdb: a database for the COVID-19 Multi-Omics Blood ATlas
Source: Nucleic Acids Res. 2022 Nov 10;51(D1):D896–905. doi: 10.1093/nar/gkac1019 (PMC9825482; doi:10.1093/nar/gkac1019)
Supplement: gkac1019_Supplemental_File [file gkac1019_supplemental_file.docx]

**SUPPLEMENTARY TABLE 1**

# COMBAT Consortium members and affiliations

David J Ahern (2), Zhichao Ai (2), Mark Ainsworth (19), Chris Allan (1), Alice Allcock (1), Brian Angus (19), M Azim Ansari (1,6), Carolina V Arancibia-Cárcamo (16, 18), Dominik Aschenbrenner (16), Moustafa Attar (1,2), J Kenneth Baillie (28), Eleanor Barnes (6,16,18,19), Rachael Bashford-Rogers (1), Archana Bashyal (19), Sally Beer (39), Georgina Berridge (5), Amy Beveridge (8), Sagida Bibi (8), Tihana Bicanic (33), Luke Blackwell (8), Paul Bowness (13,18), Andrew Brent (19,38), Andrew Brown (1), John Broxholme (1), David Buck (1), Katie L Burnham (27), Helen Byrne (7), Susana Camara (8), Ivan Candido Ferreira (3), Philip Charles (4,5), Wentao Chen (20), Yi-Ling Chen (3), Amanda Chong (1), Elizabeth A Clutterbuck (8), Mark Coles (2), Christopher P Conlon (17,19,31), Richard Cornall (25), Adam P Cribbs (13), Fabiola Curion (1), Emma E Davenport (27), Neil Davidson (19), Simon Davis (5), Calliope A Dendrou (1), Julie Dequaire (19), Lea Dib (2), James Docker (1), Christina Dold (8), Tao Dong (17,25), Damien Downes (3), Hal Drakesmith (3), Susanna J Dunachie (6,19,25,31), David A Duncan (3,21), Chris Eijsbouts (1,4), Robert Esnouf (1,4), Alexis Espinosa (39), Rachel Etherington (3,25), Benjamin Fairfax (3,10,18,19), Rory Fairhead (19), Hai Fang (1), Shayan Fassih (19), Sally Felle (8), Maria Fernandez Mendoza (39), Ricardo Ferreira (1), Roman Fischer (5,17), Thomas Foord (19), Aden Forrow (7), John Frater (6,19), Anastasia Fries (19,20), Veronica Gallardo Sanchez (19), Lucy C Garner (3,16), Clementine Geeves (1), Dominique Georgiou (39), Leila Godfrey (1), Tanya Golubchik (1,4), Maria Gomez Vazquez (2), Angie Green (1), Hong Harper (1), Heather A Harrington (1,7), Raphael Heilig (5), Svenja Hester (5), Jennifer Hill (8), Charles Hinds (34), Clare Hird (19), Ling-Pei Ho (20,25), Renee Hoekzema (7), Benjamin Hollis (12), Jim Hughes (3,26), Paula Hutton (19), Matthew A Jackson-Wood (2), Ashwin Jainarayanan (2), Anna James-Bott (13), Kathrin Jansen (2), Katie Jeffery (19), Elizabeth Jones (8), Luke Jostins (2,4), Georgina Kerr (36), David Kim (19), Paul Klenerman (6,16), Julian C Knight (1,17,18,19), Vinod Kumar (2), Piyush Kumar Sharma (3,10), Prathiba Kurupati (3,25), Andrew Kwok (1), Angela Lee (1), Aline Linder (8), Teresa Lockett (19), Lorne Lonie (1), Maria Lopopolo (1), Martyna Lukoseviciute (3), Jian Luo (20), Spyridoula Marinou (8), Brian Marsden (2,22), Jose Martinez (39), Philippa C Matthews (6), Michalina Mazurczyk (3,25), Simon McGowan (3), Stuart McKechnie (19), Helen McShane (9), Adam Mead (3,18,19), Alexander J Mentzer (1,19), Yuxin Mi (1), Claudia Monaco (2), Ruddy Montadon (1), Giorgio Napolitani (3,25), Isar Nassiri (3,10), Alex Novak (39), Darragh P O'Brien (5), Daniel O'Connor (8,18), Denise O'Donnell (19), Graham Ogg (3,18,25), Lauren Overend (1), Inhye Park (2), Ian Pavord (20), Yanchun Peng (3,17,25), Frank Penkava (13), Mariana Pereira Pinho (3,25), Elena Perez (39), Andrew J Pollard (8,18), Fiona Powrie (2), Bethan Psaila (3,18), T Phuong Quan (32), Emmanouela Repapi (3,26), Santiago Revale (1), Laura Silva-Reyes (8), Jean-Baptiste Richard (2,3), Charlotte Rich-Griffin (1), Thomas Ritter (19), Christine S Rollier (8), Matthew Rowland (19), Fabian Ruehle (11), Mariolina Salio (3,25), Stephen Nicholas Sansom (2), Raphael Sanches Peres (2), Alberto Santos Delgado (4,23,24), Tatjana Sauka-Spengler (3), Ron Schwessinger (3), Giuseppe Scozzafava (1), Gavin Screaton (1,36), Anna Seigal (7), Malcolm G Semple (29), Martin Sergeant (3), Christina Simoglou Karali (3), David Sims (3), Donal Skelly (6,14,19), Hubert Slawinski (1), Alberto Sobrinodiaz (39), Nikolaos Sousos (3,18,19), Lizzie Stafford (19), Lisa Stockdale (8), Marie Strickland (3), Otto Sumray (7,37), Bo Sun (1), Chelsea Taylor (3,10), Stephen Taylor (3), Adan Taylor (19), Supat Thongjuea (3), Hannah Thraves (39), John A Todd (1), Adriana Tomic (8), Orion Tong (3,10), Amy Trebes (1), Dominik Trzupek (1), Felicia Anna Tucci  (1), Lance Turtle (29,30), Irina Udalova (2), Holm Uhlig (16,19), Erinke van Grinsven (2), Iolanda Vendrell (5), Marije Verheul (8), Alexandru Voda (2), Guanlin Wang (3), Lihui Wang (2), Dapeng Wang (1), Peter Watkinson (14,19), Robert Watson (3,10,19), Michael Weinberger (3,35), Justin Whalley (1), Lorna Witty (1), Katherine Wray (3,19), Luzheng Xue (20), Hing Yuen Yeung (1), Zixi Yin (17,25), Rebecca K Young (19), Jonathan Youngs (33), Ping Zhang (1), Yasemin-Xiomara Zurke (2), ISARIC4C Consortium (40).

## Affiliations

1: Wellcome Centre for Human Genetics, Nuffield Department of Medicine, University of Oxford, UK

2: Kennedy Institute for Rheumatology, Nuffield Department of Orthopaedics, Rheumatology and Musculoskeletal Diseases, University of Oxford, UK

3: MRC Weatherall Institute of Molecular Medicine (WIMM), University of Oxford, UK

4: Big Data Institute, Nuffield Department of Medicine, University of Oxford, UK

5: Target Discovery Institute, Nuffield Department of Medicine, University of Oxford, UK

6: Peter Medawar Building for Pathogen Research, Nuffield Department of Medicine, University of Oxford, UK

7: Mathematical Institute, University of Oxford, UK

8: Oxford Vaccine Group, Department of Paediatrics, University of Oxford, UK

9: Department of Paediatrics, University of Oxford, UK

10: Department of Oncology, University of Oxford, UK

11: Department of Physics, University of Oxford, UK

12: Jenner Institute, Nuffield Department of Medicine, University of Oxford, UK

13: Botnar Research Centre, Nuffield Department of Orthopaedics, Rheumatology and Musculoskeletal Diseases, University of Oxford, UK

14: Nuffield Department of Clinical Neuroscience, University of Oxford, UK

15: Department of Statistics, University of Oxford, UK

16: Translational Gastroenterology Unit, University of Oxford, UK

17: Chinese Academy of Medical Science Oxford Institute (COI), University of Oxford, UK

18: NIHR Oxford Biomedical Research Centre, Oxford, UK

19: Oxford University Hospitals NHS Foundation Trust, Oxford, UK

20: Respiratory Medicine Unit, Nuffield Department of Medicine, University of Oxford, UK

21: Diamond Light Source, Didcot, UK

22: Centre for Medicines Discovery, Nuffield Department of Medicine, University of Oxford, UK

23: Center for Health Data Science, University of Copenhagen, DK

24: Novo Nordisk Foundation Center for Protein Research, University of Copenhagen, DK

25: MRC Human Immunology Unit, Radcliffe Department of Medicine, University of Oxford, UK

26: MRC WIMM Centre for Computational Biology, Radcliffe Department of Medicine, University of Oxford, UK

27: Wellcome Sanger Institute, Cambridge, UK

28: Genetics and Genomics, Roslin Institute, University of Edinburgh, Easter Bush, Midlothian, UK

29: NIHR Health Protection Research Unit in Emerging and Zoonotic Infections, Institute of Infection, Veterinary and Ecological Sciences, University of Liverpool, Liverpool

30: Tropical and Infectious Diseases Unit, Liverpool University Hospitals NHS Foundation Trust, Liverpool, UK

31: Centre for Tropical Medicine and Global Health, Nuffield Department of Medicine, University of Oxford, UK

32: Experimental Medicine Division, Nuffield Department of Medicine, University of Oxford, UK

33: Institute for Infection and Immunity, St George's University of London, UK

34: The William Harvey Research Institute, Bart’s and The London, Queen Mary University of London, UK

35: Department of Physiology, Anatomy and Genetics, University of Oxford, UK

36: Medical Sciences Division, University of Oxford, UK

37: Ludwig Institute for Cancer Research, Nuffield Department of Medicine, University of Oxford, UK

38: Nuffield Department of Medicine, University of Oxford, UK

39: Emergency Medicine Research Oxford, Oxford University Hospitals NHS Foundation Trust, Oxford, UK

40: https://isaric4c.net/about/authors/
